# Supplementary material for: Fetal-to-fetal kidney transplantation in utero
Source: Commun Biol. 2025 Mar 3;8:349. doi: 10.1038/s42003-025-07783-9 (PMC11876676; doi:10.1038/s42003-025-07783-9)
Supplement: Supplementary file 2 — Supplementary Information [file 42003_2025_7783_MOESM2_ESM.pdf]

Title

Fetal-to-Fetal Kidney Transplantation *in Utero*

Authors

Keita Morimoto[1], Shuichiro Yamanaka[1,2]*, Kenji Matsui[1], Yoshitaka Kinoshita[1,3], Yuka Inage[1,4], Shutaro Yamamoto[5], Nagisa Koda[1], Naoto Matsumoto[1], Yatsumu Saito[1], Tsuyoshi Takamura[1], Toshinari Fujimoto[1], Shohei Fukunaga[1], Susumu Tajiri[1], Kei Matsumoto[1], Katsusuke Ozawa[6], Seiji Wada[6], Eiji Kobayashi[7], Takashi Yokoo[1]*

Affiliations

1. Division of Nephrology and Hypertension, Department of Internal Medicine, The Jikei University School of Medicine, Tokyo 105-8461, Japan
2. Kidney Applied Regenerative Medicine, Project Research Units, The Jikei University School of Medicine, Tokyo 105-8461, Japan
3. Department of Urology, Graduate School of Medicine, The University of Tokyo, Tokyo 113-8654, Japan
4. Department of Pediatrics, The Jikei University School of Medicine, Tokyo 105-8461, Japan
5. Department of Urology, The Jikei University School of Medicine, Tokyo 105-8461, Japan
6. Center for Maternal-Fetal, Neonatal and Reproductive Medicine, National Center for Child Health and Development, Tokyo 157-8535, Japan
7. Department of Kidney Regenerative Medicine, The Jikei University School of Medicine, Tokyo 105-8461, Japan

* Corresponding author emails: shu.yamanaka@jikei.ac.jp, tyokoo@jikei.ac.jp

Table of Contents

Supplementary Fig. 1 | Structure of the fetal kidney before transplantation.
Supplementary Fig. 2 | Analysis of vascular chimerism between mice and rats.

Supplementary Table 1 | Transplacental tacrolimus transfer (n = 2).
Supplementary Table 2 | Creatinine clearance (CCr) of urine produced from MNBs (n = 1).
Supplementary Table 3 | List of primary antibodies.

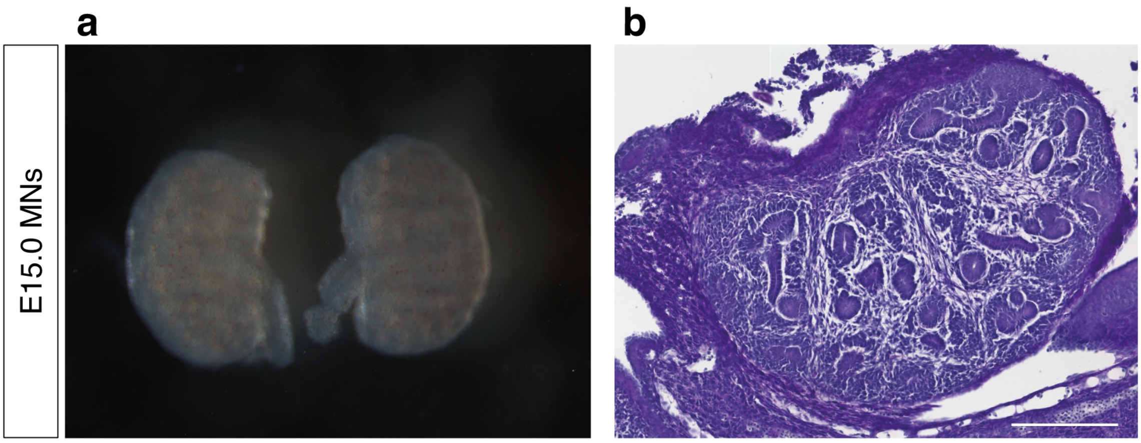

Supplementary Fig. 1 Structure of the fetal kidney before transplantation

a Structure of the fetal kidney before transplantation (*n* = 1). **b** Periodic acid-Schiff staining revealed that the fetal kidney had not formed glomeruli or renal tubules before transplantation. Scale bars, 200 μm in (**b**). MNs, metanephroi

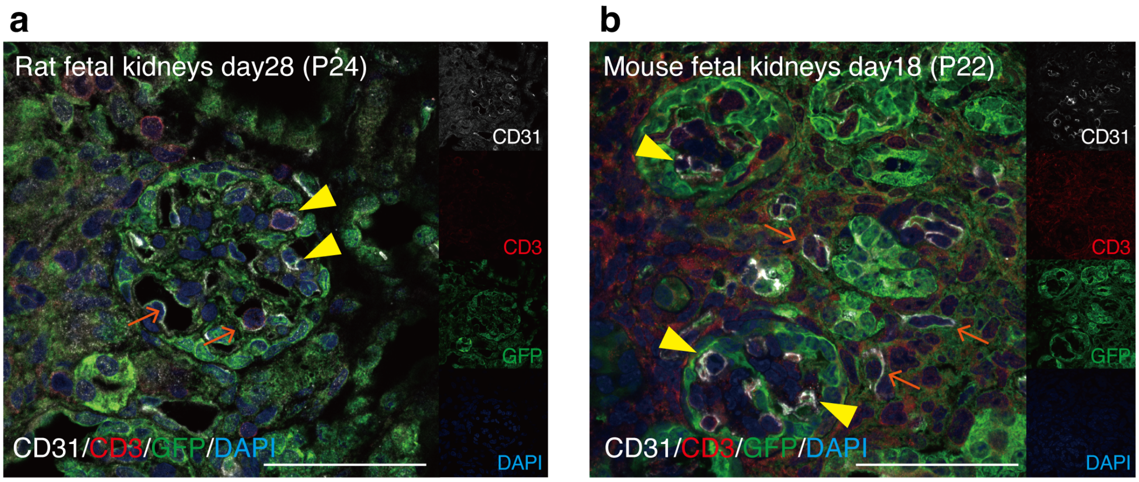

Supplementary Fig. 2 Analysis of vascular chimerism between mice and rats

a Rat fetal kidneys (E14.5) were transplanted into rat fetuses (E18.0–E18.5) and retrieved 28 days after transplantation. Yellow arrowheads point to GFP-negative/CD31-positive vessels within glomeruli, indicating recipient-derived vessels. Orange arrows highlight GFP-positive/CD31-positive vessels within glomeruli, indicating donor-derived vessels. b Mouse fetal kidneys (E13.0) were transplanted into rat fetuses (E18.0–E18.5) and retrieved 18 days after transplantation. Yellow arrowheads point to GFP-negative/CD31-positive vessels within glomeruli, indicating recipient-derived vessels. Orange arrows highlight GFP-positive/CD31-positive vessels in the interstitium, indicating donor-derived vessels. Scale bars, 50 μm (a), (b). GFP, green fluorescent protein

Supplementary Table 1. Transplacental tacrolimus transfer (*n* = 2).

Start of administration	Cesarean section, blood collection from the fetuses	Dose (mg/kg/day)	Number of fetuses	Maternal tacrolimus blood level (ng/mL) (24-h trough)	Fetal tacrolimus blood level (ng/mL) (24-h trough)	Migration rate (%)
E18.0	E21.0	0.2	12	1.57	0.65	41.4
E18.0	E21.0	1.0	11	7.59	2.7	36.4

Tacrolimus was administered subcutaneously to pregnant rats at 0.2 or 1.0 mg/kg/day every 24 h on gestational days 18–20 (E18.0–E20.0) before cesarean section.

Supplementary Table 2. Creatinine clearance (CCr) of urine produced from MNBs (*n* = 1).

Day	Urine creatinine levels [mg/dL]	Srum creatinine levels [mg/dL]	Urine storage time [h]	Urine volume [mL]	Urine volume [μL/min]	Urine volume [mL/24h]	CCr[μL/min]
70–75	13.4	0.18	116.0	4.4	0.632	0.91	47.1
75–77	13.73	0.19	45.0	2.0	0.741	1.07	53.5
77–78	12.45	0.19	24.0	1.3	0.903	1.30	59.2
78–81	13.55	0.19	51.0	2.0	0.654	0.94	46.6
81–84	14.05	0.19	92.0	3.4	0.616	0.89	45.5
84–85	11.52	0.19	24.0	1.2	0.833	1.20	50.5
85–88	14.02	0.17	72.0	2.8	0.648	0.93	53.5
88–89	11.15	0.17	24.0	0.4	0.278	0.40	18.2
89–92	14.12	0.16	78.7	4.0	0.848	1.22	74.8
92–97	14.34	0.16	116.2	3.6	0.516	0.74	46.3
97–102	17.09	0.15	122.5	4.6	0.626	0.90	71.3
102–106	15.78	0.15	96.3	3.4	0.588	0.85	61.9
106–112	18.24	0.15	147.6	5.4	0.610	0.88	74.2
112–121	20.98	0.16	213.4	6.3	0.492	0.71	64.5
121–126	17.24	0.16	191.8	3.8	0.330	0.48	35.6
126–131	18.19	0.18	138.8	4.2	0.504	0.73	51.0
131–135	17.89	0.18	104.3	3.4	0.543	0.78	54.0
135–138	14.92	0.15	59.3	4.0	1.124	1.62	111.8
138–144	12.96	0.15	143.7	4.6	0.534	0.77	46.1
144–147	17.91	0.15	72.4	3.6	0.828	1.19	98.9
147–150	16.95	0.15	74.5	3.6	0.806	1.16	91.0

This is the raw data for urine volume and creatinine clearance shown in Fig. 3d.

Supplementary Table 3. List of primary antibodies.

Antigen	Host	Supplier	Cat. No.	Dilution
Nephrin	Guinea pig	Progen	GP-N2	1:100
LTL biotin	-	Vector	B-1325	1:200
E-cadherin	Mouse	Thermo	610181	1:100
E-cadherin	Rabbit	Cell Signaling	3195S	1:100
GFP	Rabbit	MBL	MBL598	1:500
GFP	Chicken	Abcam	ab13970	1:100
CD31	Goat	R&D	AF3628	1:100
CD3	Mouse	DAKO	Clone F7.2.38	1:100
CD3	Rabbit	Abcam	ab5690	1:100

This is a list of primary antibodies used for immunofluorescence and immunohistochemistry.
