# Supplementary material for: Fetal-to-fetal kidney transplantation in utero
Source: Commun Biol. 2025 Mar 3;8:349. doi: 10.1038/s42003-025-07783-9 (PMC11876676; doi:10.1038/s42003-025-07783-9)
Supplement: Supplementary file 3 — Description of Additional Supplementary Files [file 42003_2025_7783_MOESM3_ESM.docx]

**Description of Additional Supplementary Files**

Supplementary Date 1

Figure 2a Neonates body weght

This is the raw data for Figure 2a, showing the body weight gain between transplanted (n = 3) and non-transplanted (n = 3) neonates after natural delivery on embryonic day 22.

Supplementary Date 2

Figure 2b MNBs Long diameter

This is the raw data for Figure 2b, showing the temporal change in the long diameter of GFP-positive tissue (MNBs) observed on the body surface of the neonates (n = 4).

Supplementary Movie 1 | Movie of fetal-to-fetal transplantation.

The uterine wall was punctured with the 15–16G needle loaded with GFP rat MNBs on the dorsal side of the fetus, and the needle was further inserted into the fetal subcutaneous space while being monitored under a stereomicroscope. The needle was advanced approximately 5 mm under the fetal skin, and 0.1–0.5 mL of Hank’s balanced salt solution (HBSS, TERUMO, Tokyo, Japan) was ejected to transplant the GFP rat MNBs loaded onto the needle tip into the fetal subcutaneous space. Immediately after transplantation, the needle was withdrawn.

Supplementary Movie 2 | Movie of urine aspiration by puncture.

A single puncture was performed on the back of the animals using a 23–29G needle, and urine produced from MNBs was aspirated.
